# Differential Dynamics of Humoral and Cell-Mediated Immunity with Three Doses of BNT162b2 SARS-CoV-2 Vaccine in Healthcare Workers in Japan: A Prospective Cohort Study

**DOI:** 10.3390/vaccines10071050

**Published:** 2022-06-30

**Authors:** Keita Yamashita, Akira Suzuki, Shiori Takebayashi, Akihiro Toguchi, Kenya Ogitani, Noriyasu Niizeki, Osanori Nagura, Kazuki Furuhashi, Moriya Iwaizumi, Masato Maekawa

**Affiliations:** Department of Laboratory Medicine, Hamamatsu University School of Medicine, Hamamatsu 431-3192, Japan; suzukia@hama-med.ac.jp (A.S.); tshiori@hama-med.ac.jp (S.T.); atoguchi@hama-med.ac.jp (A.T.); k.ogi@hama-med.ac.jp (K.O.); niizeki@hama-med.ac.jp (N.N.); rikyo@hama-med.ac.jp (O.N.); k.furu@hama-med.ac.jp (K.F.); iwaizumi@hama-med.ac.jp (M.I.)

**Keywords:** SARS-CoV-2, BNT162b2 vaccine, humoral immunity, cell-mediated immunity, anti-RBD antibody, ELISpot-, antibody avidity, T-SPOT, harmonize

## Abstract

Vaccines against SARS-CoV-2 with good efficacy are now available worldwide. However, gained immunity diminishes over time. Here, we investigate the course of both humoral and cell-mediated immunity in response to three doses of the Pfizer mRNA BNT162b2 SARS-CoV-2 vaccine in healthcare workers in Japan. SARS-CoV-2 anti-receptor-binding domain (RBD) antibodies (total Ig, IgG), neutralizing antibodies (NAb), and ELISpot were measured in serum and whole blood samples collected after each vaccine dose. ELISpot numbers were higher than the cutoff values in most participants at all times. It was suggested that the difference in behavior between humoral immunity and cell-mediated immunity with age is complementary. Anti-RBD total Ig, IgG, and NAb indicated a high correlation at each time point after vaccine doses. Total Ig was retained long-term after the second dose and increased significantly faster by the booster dose than IgG. Nab levels of all subjects were ≤20% six months after the second dose, and the correlation coefficient was greatly reduced. These are due to the avidity of each antibody and differences among commercial kits, which may affect the evaluation of immunokinetics in previous COVID-19 studies. Therefore, it is necessary to harmonize reagents categorized by the same characteristics.

## 1. Introduction

Over two years have passed since the onset of the coronavirus disease-2019 (COVID-19) epidemic, and effective vaccines are now available. In particular, the efficacies of two mRNA vaccines, BNT162b2 (Pfizer-BioNTech, Mainz, Germany) and mRNA-1273/TAK-919 (Moderna, USA), have been reported to be 95% [1,2]. However, it is thought that the antibody titer peaks after two doses of the SARS-CoV-2 vaccine. It then starts to diminish, and after six months, it decreases considerably [3]. In this context, as breakthrough infections of COVID-19 are on the rise—several concerning variants (e.g., Alpha (B.1.1.7), Beta (B.1.351), Delta (B.1.617.2), and Omicron (B.1.1.529)) have emerged—three doses and provision of effective treatment will be essential [4,5,6].

The efficacy of the mRNA vaccine is believed to be due not only to the antibody titer, but also humoral and cell-mediated immunity [3,7]. However, the interval for measuring immunity is quite large, and the exact mechanisms involved over six months post vaccine dose are not well understood. In Japan, mass vaccination campaigns started in February 2021, with the third (booster) dose recommended since the end of 2021. Long-term data on the kinetics of the humoral and cell-mediated immune responses remain scarce in the literature. As neutralization antibody titers are not easily and routinely analyzed in the clinical laboratory, other methods need to be explored. In this regard, the use of automated analyzers to measure levels of anti-receptor-binding domain (RBD) antibodies may be an effective means of understanding the state of humoral immunity. However, in previous studies for COVID-19, immune levels depending on the characteristics of the antibody measuring reagent used may be due to differences in evaluation of immunokinetics.

In addition, it is not clear what causes the differences in the behavior of humoral and cell-mediated immune responses between individuals after receiving the booster dose. It is reported that gender, age, obesity, and medical history are involved in the peak and subsequent decay of antibody titers after the second dose [8]. Clarifying the course of immune behavior stimulated by the booster dose will provide useful information for future infection control.

In this study, to investigate the long-term dynamics of humoral and cell-mediated immunity after mRNA vaccine targeting RBD of wild-type SARS-CoV-2, we analyzed the levels of parameters indicating immunity against SARS-CoV-2 in the peripheral blood of hospital healthcare workers by three doses of the BNT162b2 vaccine in Japan. The anti-RBD IgG, anti-RBD total Ig, and neutralizing antibodies were used to assess humoral immunity and for comparison of assays from different suppliers.

## 2. Materials and Methods

### 2.1. Study Cohort, Design, and Sample Processing

Health care workers from the Hamamatsu University School of Medicine who had received two doses of the BNT162b2 vaccine (Comirnaty 30 mg, Pfizer-BioNTech, USA) injected intramuscularly into the deltoid muscle at three weekly intervals were recruited (the first dose was administered in March 2021). A total of 50 healthy volunteer healthcare workers (31 females and 19 males, median age 44 years) were enrolled in this prospective cohort study.

Participants received the first dose of the vaccine in March, followed by a second dose 21 days later. The subjects’ information, including age and gender, was obtained. To assess the subjective perception of post-vaccine dose reactions, medical check sheets, including the following typical categories, were collected: (1) fever (≥38 °C); (2) pain at the injection site; (3) headache; and (4) fatigue/tiredness. For categories 2 to 4, the intensity of symptoms was expressed on a self-assessed scale of 1 to 3. Furthermore, according to the vaccination campaign, the booster dose was administered in January 2022; thus, we continued and included additional follow-up studies.

Venous blood was collected on 11 occasions; before vaccine dose, 3 days, and 2 weeks after the first dose; 3 days, 2 weeks, and 1, 2, 4, and 6 months after the second dose. Additional blood collection was performed before the booster dose (10 months after two vaccine doses), and 2 weeks after the booster dose. Sera were obtained by centrifugation for 15 min at 1500× *g* at 20 ± 3 °C. Most analyses, including routine biochemistry, immunochemistry, and detection of immunity against SARS-CoV-2, were performed on the same day. The remaining sera were stored at −80 °C until use. Whole blood was used for routine hematology tests and T-SPOT assays to assess cell-mediated immunity. The full study schedule is presented in Table S1.

### 2.2. Detection of SARS-CoV-2 Specific Antibodies

This study investigated the levels of SARS-CoV-2 anti-RBD antibodies. The use of antibodies against spike proteins and nucleocapsids was designed to reflect the nature of a SARS-CoV-2 infection. Antibody responses were analyzed using Elecsys^®^ Anti-SARS-CoV-2S assay (anti-RBD total immunoglobulin [Ig]) and Elecsys^®^ Anti-SARS-CoV-2 antibodies assay kit (anti-nucleocapsids total Ig) on a cobas^®^ 8000 e801 module (Roche Diagnostics, Rotkreuz, Switzerland), using a double-antigen sandwich test principle and based on electrochemiluminescence immunoassays [9,10]. The anti-RBD total Ig allowed for the quantitative detection of total Ig (predominantly IgG) aimed at the SARS-CoV-2 spike protein receptor-binding domain. The measurement threshold was 0.4 U/mL, and values >0.8 U/mL were considered positive. According to the manufacturer’s protocol, samples with a titer >250 U/mL were serially diluted tenfold until a titer of 250 U/mL was reached. Furthermore, in this study, the binding antibody unit (BAU) conversion value was adopted using the alignment coefficient (Roche; 1 U/mL = 1.029 BAU/mL) by a harmonization program using the WHO international standard material, NIBSC code 20/136, for anti-SARS-CoV-2 immunoglobulin [11]. Elecsys^®^ Anti-SARS-CoV-2 antibodies assay kit is intended for the qualitative detection of antibodies to a recombinant protein representing the nucleocapsid (N) antigen. The assay results were interpreted as follows: cutoff index < 1.0 for samples nonreactive/negative for anti-N antibodies; cutoff index ≥ 1.0 for samples reactive/positive for anti-N antibodies.

The second system measured two anti-SARS-CoV-2 antibody levels (HISCL^TM^ anti-S1-IgG and anti-N-IgG) using a two-step sandwich immunoassay principle and an automated high-sensitivity chemiluminescence enzyme immunoassay system (HISCL^TM^-5000; Sysmex Corporation, Kobe, Japan) [12]. Levels of anti-RBD IgG titers were expressed in BAU/mL and evaluated in relation to a cutoff index calculated by the quantification of standard anti-SARS-CoV-2 RBD antibody samples. The cutoff value was determined to be 20 BAU/mL using the Anti-SARS-CoV-2 Verification Panel for Serology Assays (NIBSC code: 20/B770) (Sysmex Guidance Document).

### 2.3. Detection of Neutralizing Antibodies against SARS-CoV-2

One function of an antibody is its neutralizing action against foreign substances (e.g., antigens). SARS-CoV-2 binds to and invades the host cell receptor human ACE2 (hACE2) via its spike protein RBD [13]. Neutralizing antibodies (NAb) that measure this binding inhibition can directly assess the protection against SARS-CoV-2 infection. In this context, NAb were analyzed using SARS-CoV-2 Neutralization Antibody Detection Kit (MBL Bio, Tokyo, Japan). The kit was designed to examine neutralization titers with a newly developed bead/cell-based Spike-ACE2 inhibition assay [14]. From the average of the duplicate readings of positive control and sample, the inhibition rate of each sample was calculated using equation 1. Dilution buffer was used as a blank, i.e., 0% inhibition control.

(1) $$\mathrm{inhibition}\;\mathrm{rate}\;(\%)=(1-\frac{\mathrm{absorbance}\;\mathrm{of}\;\mathrm{sample}}{\mathrm{absorbance}\;\mathrm{of}\;\mathrm{blank}})\times 100$$

As there are few previous studies establishing the cutoff value for the neutralizing ability using this assay, it was set as the mean plus two standard deviations calculated from the 50 subjects’ inhibition rates (%) before the vaccine dose.

### 2.4. Detection of Cell-Mediated Immunity against SARS-CoV-2

Cell-mediated immunity was measured by T-cell enzyme-linked immunospot (ELISpot) as previously described [15]. Peripheral blood mononuclear cells (PBMCs) were isolated from a whole blood sample immediately after blood collection, using Leucosep tubes (Greiner Bio-One GmbH, Mühlkreis, Austria). If PBMCs could not be separated on the day after blood collection, the T-Cell *Xtend* reagent (Oxford Immunotec Ltd., Abingdon, UK) was added to the blood collection tube, and PBMCs were then separated within 48 h. After quantification and dilution of recovered cells by GIBCO AIM V^®^ Medium (Thermo Fisher Scientific, Inc, MA, USA), 250,000 ± 50,000 PBMCs were plated into each well of a T-SPOT^®^ Discovery SARS-CoV-2 plate (T-SPOT; Oxford Immunotec Ltd., Abingdon, UK). The kit is designed to measure responses to three different, but overlapping, peptide pools to cover the protein sequences of three different SARS-CoV-2 antigens-spike glycoproteins (S1 subunit), nucleocapsid protein (enclosing RNA), and membrane protein. In addition, it provides a measure of the response to a mixture of epitopes with high genetic homology to endemic human coronaviruses, without HLA restriction, and includes negative and positive controls. Cell suspensions were incubated at 37 °C and 5% CO_2_ for 20 h, and interferon-γ secreting T cells were then detected. The T-SPOT count per well (spots/2.5 × 10^5^/well) was automatically measured using the Immunospot S6 Universal Analyzer (ELISPOT; Cellular Technology Limited, Shaker Heights, OH, USA). Subjects whose negative and positive controls did not meet the criteria of ≤10 spots and ≥20 spots, respectively, were excluded. Two of the 50 subjects in this study showed a non-specific response with >10 spots of a negative control (no specific antigen stimulation), so the remaining 48 subjects were used for the analysis. After correction by the negative control, according to the manufacturer’s protocol, the cutoff value for a positive result was ≥8 spots [16,17].

### 2.5. Other Laboratory Testing

Routine laboratory tests were performed using Hitachi LABOSPECT 008 α and 006 (Hitachi High-Tech Co., Hitachi, Japan) for biochemical assays, Cobas^®^ 8000 e801 module (Roche Diagnostics, Rotkreuz, Switzerland), and HISCL^TM^-5000 (Sysmex Corporation, Kobe, Japan) for immunochemical assays, and XN-3100 (Sysmex Corporation, Kobe, Japan) for hematology assays. The test items are shown in Table S2.

### 2.6. Statistical Analysis

All statistical analyses were performed using ‘EZR’ (Easy R) [18]. The data from 11 time points from 50 subjects are presented as the median and interquartile range (IQR). The measured values at each time point of the immunokinetic parameters (anti-RBD antibodies, NAb, and T-SPOT) were compared by age and gender using the Mann–Whitney *U* test. The correlation between specific antibodies, NAb, T-SPOT, and age was calculated using Spearman’s rank correlation coefficient. In addition, factors affecting the immune behavior response to the booster dose were investigated using multiple logistic regression analysis. The NAb data obtained two weeks after the booster dose were divided into high or low groups by the median (n = 50). Subsequently, the association analysis was performed for comparison between two groups by the immunokinetic parameters and laboratory testing at 11 time points. Finally, we collected the factors that showed significant association, including gender and age, and performed multivariate analysis. The significant level of both Mann–Whitney *U* test and the multiple logistic regression analysis was established at *p* < 0.05, and Spearman’s rank correlation coefficients was established at *p* < 0.01.

## 3. Results

### 3.1. Demographic and Clinical Data of the Study Participants

All enrolled volunteers who gave written informed consent were included in this study. The participants included 31 females (median age, 48 years; IQR 32–56 years) and 19 males (median age, 42 years; IQR 35–45 years) (Table 1). The individuals had no previous history of SARS-CoV-2 infection, as self-reported. Furthermore, the initial blood samples collected for this study were tested for the presence of anti-nucleocapsid total Ig and IgG using two types of measurement reagents to further identify and exclude possible natural infection. All serum samples tested were seronegative for the anti-nucleocapsid antibody.

### 3.2. Time Course of SARS-CoV-2 Anti-RBD Antibodies after COVID-19 Vaccination

The time course of anti-RBD total Ig and IgG from vaccine dose was plotted by gender and age (two groups, above or below 45 years old by dividing by the median of all subjects in this study). A graph showing the time course of each subject is shown in Figure S1a–d. In most cases, the antibody titer peaked two weeks after the second dose. The titer then started to wane, but anti-RBD IgG waned at a faster rate than the anti-RBD total Ig.

**Figure 1:**
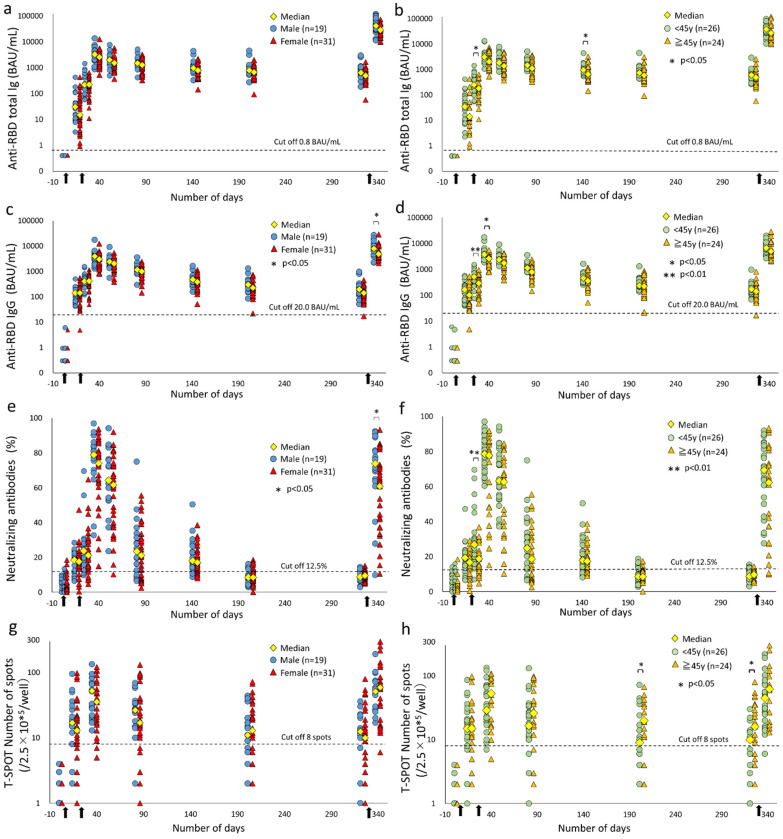
(**a**–**h**). A one-year dynamics of humoral and cell-mediated immunity against SARS-CoV-2 with three doses of the BNT162b2 vaccine. The levels of anti-RBD total Ig (**a**,**b**) and IgG (**c**,**d**), neutralizing antibodies (**e**,**f**), and T-SPOT (**g**,**h**) against SARS-CoV-2 are plotted in a time series after vaccine dose by gender and age. Blue and red plots represent the results by gender. Green and orange plots represent the results by age in two groups over or under 45 years old. The yellow plots represent the median of each time point in the group. The dashed line represents the cutoff value in each immunity parameter. *p*-values were calculated using the Mann–Whitney U test, and significance was set at 0.05. The arrow (black) indicates the vaccine dose date (the first, second, and booster doses were injected in March 2021, April 2021, and January 2022, respectively). Y-axis: (**a**,**b**), anti-RBD total Ig (Elecsys^®^ anti-SARS-CoV-2 S; Roche); (**c**,**d**), anti-RBD IgG (HISCL^TM^ SARS-CoV-2 S-IgG; Sysmex); (**e**,**f**), neutralizing antibodies (SARS-CoV-2 Neutralization Antibody Detection Kit; MBL); (**g**,**h**), T-SPOT (T-SPOT^®^ Discovery SARS-CoV-2; Oxford Immunotec). The anti-RBD antibodies and T-SPOT are shown on a log scale.

**Figure 2:**
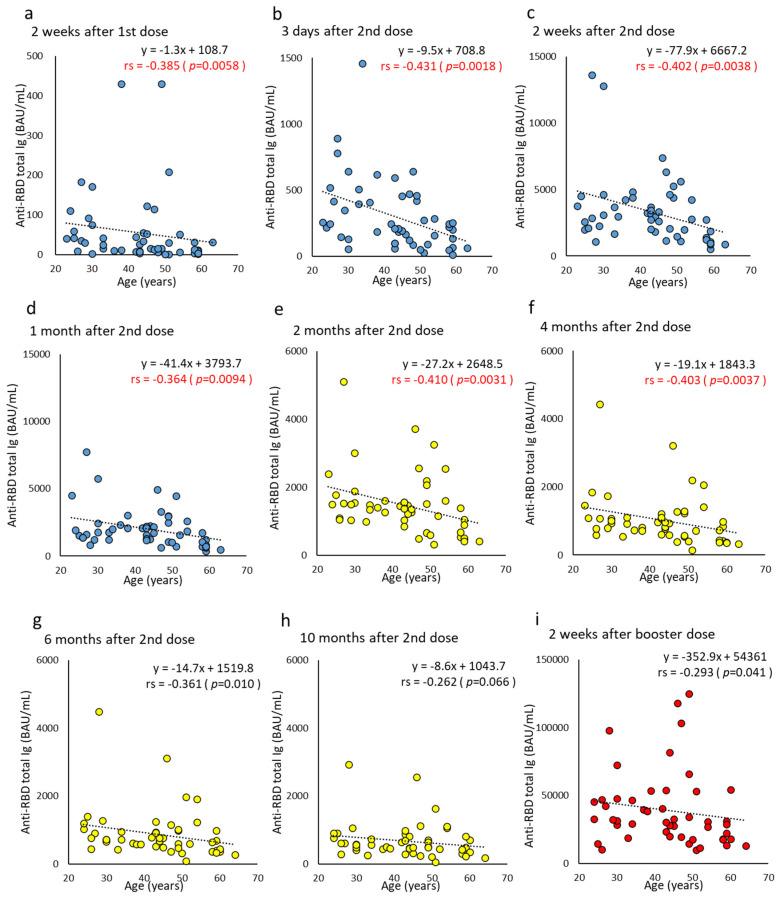
(**a**–**i**). Correlation at time points between age and anti-RBD total Ig after BNT162b2 vaccine doses. Parameter levels indicating anti-RBD total Ig of humoral immunity against SARS-CoV-2 are plotted and compared according to age at each time point post-vaccine doses. Time points until one month after two doses are colored blue (**a**–**d**), from two months to 10 months after two doses are colored yellow (**e**–**h**), and two weeks after the booster dose are colored red (**i**). *p*-values were calculated using Spearman’s rank correlation test and significance was set at 0.01. Red values indicate a significant correlation (rs, Spearman’s rank correlation coefficient).

Anti-RBD total Ig in the group under 45 years old (<45 years) was significantly higher than in the group over 45 years old (≥45 years) at three days (antibody titer median: <45 years, 251.6 BAU/mL vs. ≥45 years, 180.6 BAU/mL; *p* = 0.034) and four months post the second doses (antibody titer median: <45 years, 964.7 BAU/mL vs. ≥45 years, 653.4 BAU/mL, *p* = 0.046) (Figure 1a,b). Consistent with this finding, a negative correlation was observed between age and anti-RBD total Ig from two weeks after the first dose to four months after the second dose (correlation coefficient, −0.364 to −0.431) (Figure 2). The anti-RBD total Ig at 10 months after the second dose retained a median of 668.2-fold (IQR 518.4–1003.3) that of the cutoff value. The median anti-RBD total Ig at two weeks post the booster dose was 61.1-fold (IQR 37.1–81.9) the median before the booster dose. Compared to the same time point after the second dose, the median increase was 12.3-fold (IQR 7.6–16.2).

**Figure 3:**
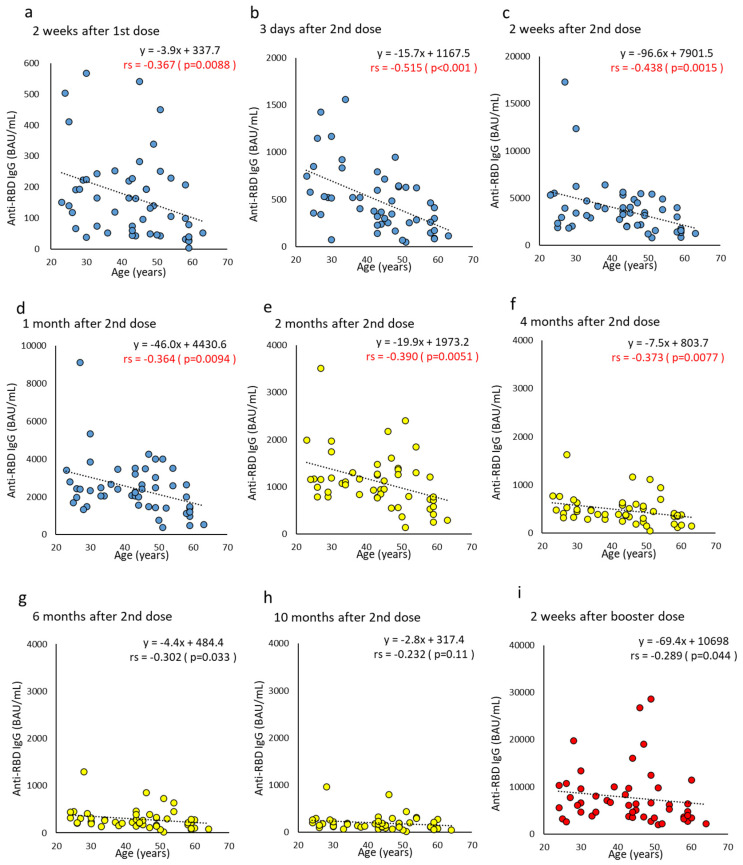
(**a**–**i**). Correlation at time points between age and anti-RBD IgG after BNT162b2 vaccine doses. Parameter levels indicating anti-RBD IgG of humoral immunity against SARS-CoV-2 are plotted and compared according to age at each time point post-vaccine doses. Time points until one month after two doses are colored blue (**a**–**d**), from two months to 10 months after two doses are colored yellow (**e**–**h**), and two weeks after the booster dose are colored red (**i**). *p*-values were calculated using Spearman’s rank correlation test and significance was set at 0.01. Red values indicate a significant correlation (rs, Spearman’s rank correlation coefficient).

The anti-RBD IgG titer at 10 months after the second dose retained a median 8.1-fold (IQR 5.6–11.4) higher than the cutoff value. Regarding gender differences, a statistically significantly higher titer of anti-RBD IgG in males was observed at two weeks after the booster dose (median antibody titer: male, 7930.5 BAU/mL vs. female, 4783.6 BAU/mL; *p* = 0.023). Furthermore, anti-RBD IgG in the <45 years showed a significantly higher titer at three days (antibody titer median: <45 years, 524.0 BAU/mL vs. ≥45 y, 292.3 BAU/mL; *p* = 0.0085) and two weeks (antibody titer median: <45 y, 3778.7 BAU/mL vs. ≥45 y, 2615.6 BAU/mL; *p* = 0.017) after the second dose (Figure 1c,d). A negative correlation with age was also observed, similar to anti-RBD total Ig (correlation coefficient, −0.364 to −0.515) (Figure 3). At two weeks after the booster dose, the median anti-RBD IgG titer was 41.2-fold (IQR 25.3–56.2) that of the baseline measurement obtained before the booster dose, and 1.8-fold (IQR 1.2–3.0) that of two weeks after the second dose. The overall increase in anti-RBD IgG titer due to vaccine dose was small in comparison with the anti-RBD total Ig.

### 3.3. Time Course of SARS-CoV-2 Neutralizing Antibodies after COVID-19 Vaccination

From the measured values before the vaccine dose, the reference value of NAb in the present assay was set to 12.5%. NAb showed similar fluctuations to both anti-RBD antibodies, peaking at two weeks after the second dose and then declining rapidly. Long-term from six to 10 months after the second dose, both anti-RBD antibodies exceeded the cutoff value in all subjects, whereas NAb exceeded it only in 10–14 subjects (20–28%) (Figure 1e,f and Figure S1e,f). NAb levels also recovered significantly with the booster dose but were found to vary among subjects with a 40% coefficient of variation (CV) compared to the peak after the second dose (26% CV).

**Figure 4:**
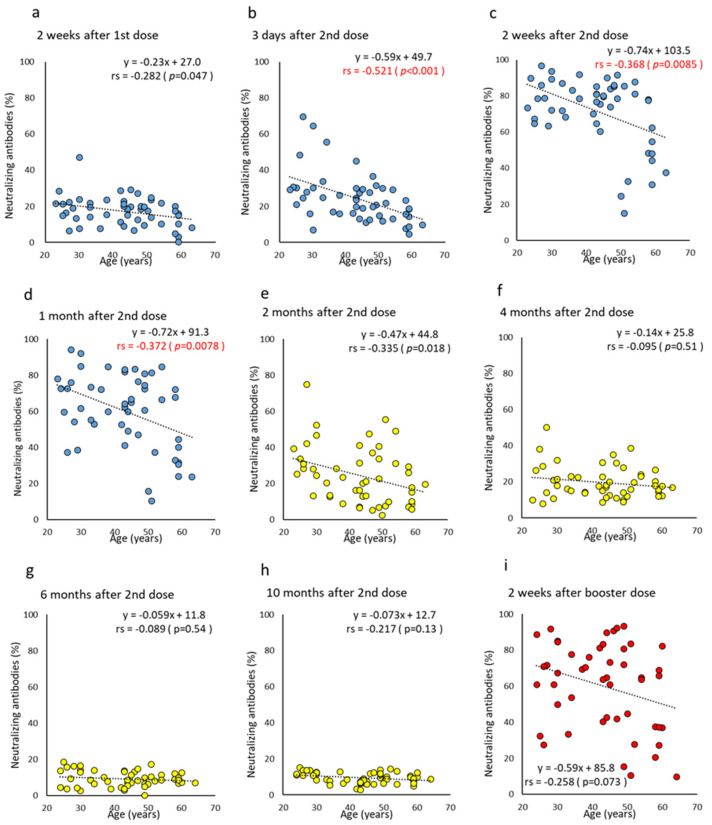
(**a**–**i**). Correlation at time points between age and neutralizing antibodies after BNT162b2 vaccine doses. Parameter levels indicating neutralizing antibodies of humoral immunity against SARS-CoV-2 are plotted and compared according to age at each time point post-vaccine doses. Time points until one month after two doses are colored blue (**a**–**d**), from two months to 10 months after two doses are colored yellow (**e**–**h**), and two weeks after the booster dose are colored red (**i**). *p*-values were calculated using Spearman’s rank correlation test and significance was set at 0.01. Red values indicate a significant correlation (rs, Spearman’s rank correlation coefficient).

For gender and age differences, a statistically significant higher NAb titer was shown at two weeks after the booster dose in males (median antibody titer: male, 74.0% vs. female, 60.9%; *p* = 0.031) and three days after the second dose in the <45 y (median NAb: <45 y, 26.9% vs. ≥45 y, 18.8%; *p* = 0.0033) (Figure 1e,f and Figure S1e,f). Similar to the anti-RBD antibodies, a negative correlation was observed from three days to one month after the second dose (correlation coefficient, −0.368 to −0.521) (Figure 4). Overall, differences in NAb titers were observed according to age, but not by gender.

### 3.4. Correlation of Each Humoral Immunity Parameter for Different Time Points after Vaccine Doses

**Figure 5:**
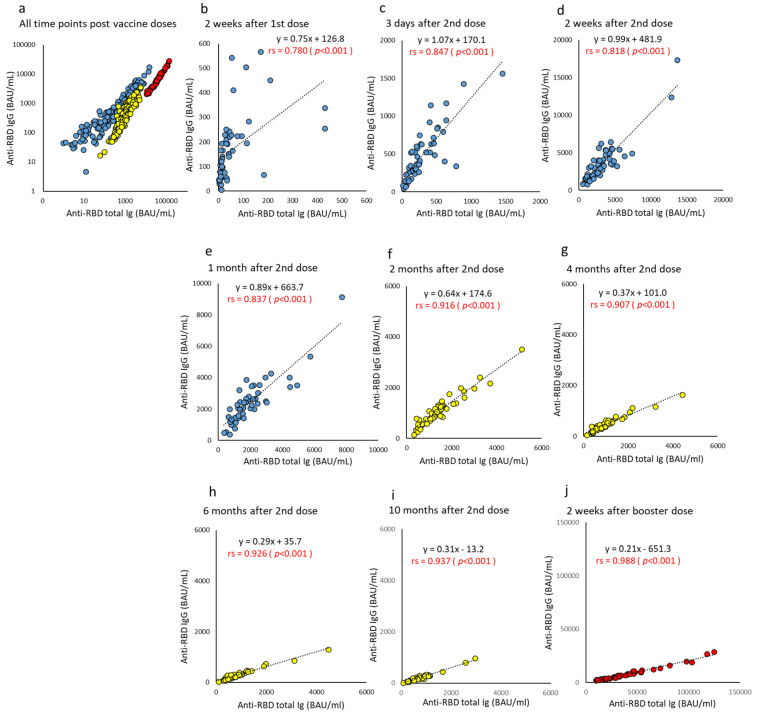
(**a**–**j**). Correlation at time points between anti-RBD total Ig and IgG after BNT162b2 vaccine doses. The levels of parameters indicating anti-RBD total Ig and IgG of humoral immunity are plotted, and the correlation is compared using all or each time point post-vaccine doses. Antibody titers at all time points are indicated on a log scale (**a**). Time points until one month after two doses are represented by blue (**b**–**e**), from two months to 10 months after two doses are represented by yellow (**f**–**i**), and two weeks after the booster dose are represented by red (**j**). *p*-values were calculated using Spearman’s rank correlation test, and significance was set at 0.01. Red values indicate a significant correlation (rs, Spearman’s rank correlation coefficient).

The scatter plot of anti-RBD total Ig and IgG at the overall time points was color-coded in three groups (Figure 5). The correlation coefficient between anti-RBD total Ig and IgG at each time series color-coded by three doses was extremely high, and the slope of the regression equation changed with time after the vaccine dose. Two weeks after the first dose, anti-RBD IgG levels rose. In contrast, levels of anti-RBD total Ig stagnated or rose slowly in many subjects. However, after the second dose, the correlation coefficient between the two antibodies gradually increased, and the slope of the regression equation gradually became small. Specifically, the correlation coefficient and slope of the regression equation were 0.847–0.937 and 1.07–0.31, respectively, from two weeks to 10 months after the second dose. Furthermore, after the booster dose, the levels of both antibodies increased significantly, but the degree of increase was greater in anti-RBD total Ig than IgG, and the angle of the slope remained small or rather decreased (correlation coefficient 0.988 and slope 0.21) (Figure 5j).

**Figure 6:**
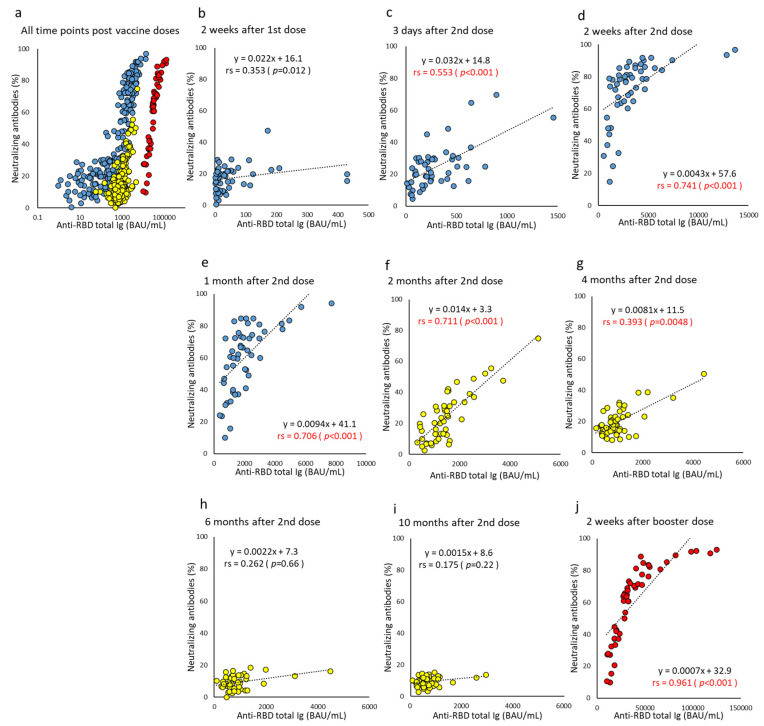
(**a**–**j**). Correlation at time points between anti-RBD total Ig and neutralizing antibodies after BNT162b2 vaccine doses. The levels of parameters indicating anti-RBD total Ig and neutralizing antibodies of humoral immunity are plotted, and the correlation is compared using all or each time point post-vaccine doses. Antibody titers at all time points are indicated on a log scale (**a**). Time points until one month after two doses are represented by blue (**b**–**e**), from two months to 10 months after two doses are represented by yellow (**f**–**i**), and two weeks after the booster dose are represented by red (**j**). *p*-values were calculated using Spearman’s rank correlation test, and significance was set at 0.01. Red values indicate a significant correlation (rs, Spearman’s rank correlation coefficient).

**Figure 7:**
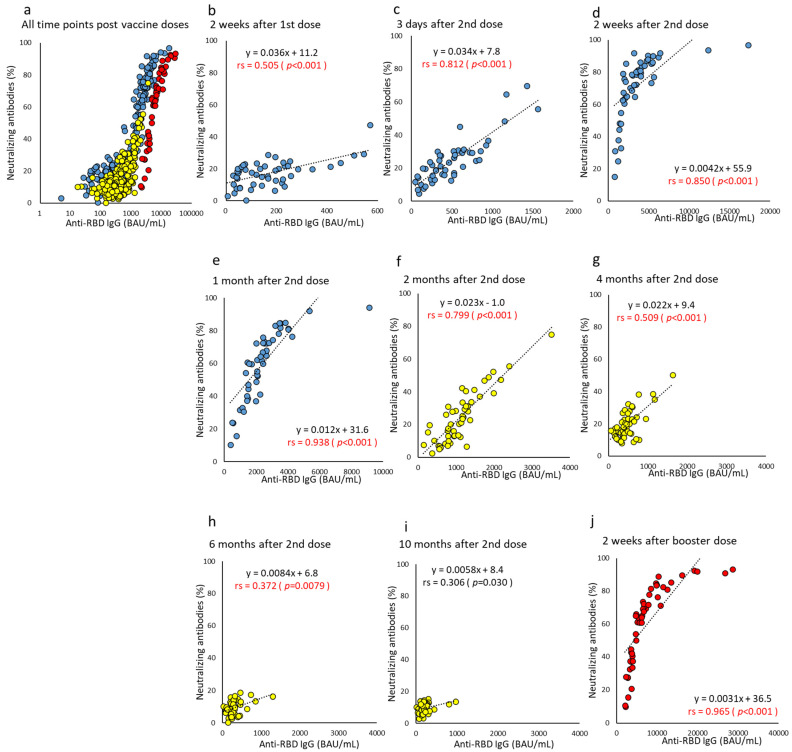
(**a**–**j**). Correlation at time points between anti-RBD IgG and neutralizing antibody after BNT162b2 vaccine doses. The levels of parameters indicating anti-RBD IgG and neutralizing antibodies of humoral immunity are plotted, and the correlation is compared using all or each time point post-vaccine doses. Antibody titers at all time points are indicated on a log scale (**a**). Time points until one month after two doses are represented by blue (**b**–**e**), from two months to 10 months after two doses are represented by yellow (**f**–**i**), and two weeks after the booster dose are represented by red (**j**). *p*-values were calculated using Spearman’s rank correlation test, and significance was set at 0.01. Red values indicate a significant correlation (rs, Spearman’s rank correlation coefficient).

Correlation between NAb and each of the anti-RBD antibodies improved after the second dose, and the correlation coefficients peaked from three days to two months after the second dose (correlation coefficient for anti-RBD total Ig, 0.553–0.741 and for anti-RBD IgG, 0.799–0.938) (Figure 6 and Figure 7). Overall, the strongest correlation was observed at all time points between the NAb and anti-RBD IgG. NAb levels are reported as a percentage; after six months, all subjects’ NAb levels were ≤20%, and the correlation was greatly reduced. However, NAb recovered to high levels after the booster dose, and the correlation between NAb and each of the anti-RBD antibodies increased significantly (correlation coefficient for anti-RBD total Ig, 0.961 and for anti-RBD IgG, 0.965).

### 3.5. Cell-Mediated Immunity, T-SPOT Numbers, and Relation with Humoral Immunity Parameters

The variation in the T-SPOT numbers was massive, with 73–103% CV. Based on our findings, the time point of the T-SPOT number peak fell within a wide range. In addition, T-SPOT numbers decreased rapidly after the peak, becoming considerably lower after two months, and then gradually decreasing again. However, T-SPOT numbers observed in most of the subjects were higher than the cutoff value (≥8) even after six or 10 months (32–36 subjects, 67–75%) (Figure 1g,h and Figure S1g,h).

**Figure 8:**
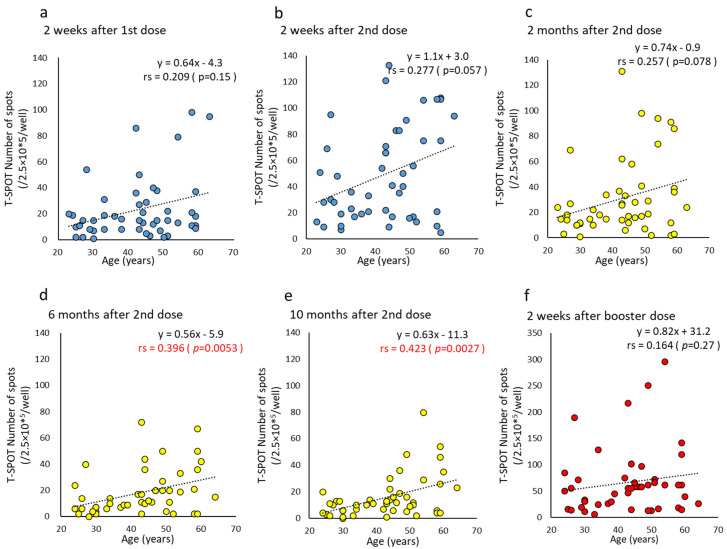
(**a**–**f**). Correlation at time points between age and T-SPOT after BNT162b2 vaccine doses. Parameter levels indicating T-SPOT of cell-mediated immunity against SARS-CoV-2 are plotted and compared according to age at each time point post-vaccine doses. Time points until two weeks after two doses are colored blue (**a**,**b**), from two months to 10 months after two doses are colored yellow (**c**–**e**), and two weeks after the booster dose are colored red (**f**). *p*-values were calculated using Spearman’s rank correlation test and significance was set at 0.01. Red values indicate a significant correlation (rs, Spearman’s rank correlation coefficient).

The T-SPOT number was statistically higher in the ≥45 y than in the <45 y (T-SPOT median: <45 y, 9–10/2.5 × 10^5^/well; ≥45 y, 16–20/2.5 × 10^5^/well, *p* = 0.022–0.032). Interestingly, age and T-SPOT number showed a positive correlation in the opposite direction to humoral immunity parameters from six to 10 months after the second dose (correlation coefficient, 0.396–0.423) (Figure 8).

The T-SPOT number at two weeks after the booster dose was a median 4.1-fold (IQR 2.6–7.2) that of the median before the booster doses and 1.4-fold (IQR 0.8–2.0) that of two weeks after the second dose. Furthermore, the T-SPOT numbers recovered significantly in individuals who originally had high immunity, whereas those who originally had low immunity responded slowly and their T-SPOT numbers remained low. The T-SPOT number of classic coronavirus proteins and membrane and nucleocapsid proteins of SARS-CoV-2 did not show a particular trend in any group. The correlation between T-SPOT numbers and antibody titers (anti-RBD total Ig and IgG, NAb) was low at all time points, and no significant correlation was observed (Figures S2–S4).

### 3.6. Factors Affecting the Neutralizing Antibody Peak by the Third Dose

Despite the correlation between the two anti-RBD antibodies, there were statistically significant differences by gender and age for each time point. Therefore, a multivariate logistic regression analysis was performed dividing NAb levels into two groups (NAb; <65%, ≥65%), with the NAb median (n = 50) at two weeks after the booster dose. Therefore, no association was found between NAb levels after the booster dose and the gender or age. Conversely, a strong association was observed between the NAb ≥ 65% group and the anti-RBD antibodies at the peak after the second dose. Of the two anti-RBD antibodies, total Ig was more relevant than IgG in the NAb ≥ 65% group. Furthermore, the T-SPOT number at six months after the second dose was higher in the NAb ≥ 65% group than in the NAb < 65% group (Table 2).

In relation to clinical laboratory test values, a significant decrease in the eosinophil count and a significant increase in the red blood cell count were observed in the NAb ≥ 65% group (Table 2). The decrease in each immune parameter from a peak after the second dose was greater in anti-RBD total Ig and the NAb in the NAb ≥ 65% group (Table S3).

## 4. Discussion

The humoral immunity (Roche anti-RBD total Ig antibodies; Sysmex anti-RBD IgG; NAb) and cell-mediated immunity (T-SPOT) of each subject were evaluated over one year using the same analytical methods. Immune responses to SARS-CoV-2 vary owing to factors such as a history of diabetes, administration of immunosuppressive drugs, dietary habits including alcohol consumption, the time interval from the first dose to the second dose, and incidence of severe COVID-19 infection [19,20,21]. Our prospective cohort study was conducted by narrowing the subjects to healthcare workers based at one facility with relatively similar behavioral histories in the same area and with tightly controlled vaccine dose intervals. During the one-year follow-up, anti-nucleocapsid antibodies were not elevated in any subject, indicating no infections with COVID-19.

Roche’s anti-RBD total Ig was measured by an automated analyzer equipped with an electrochemiluminescence immunoassay analyzer (ECLIA) based on the double-antigen sandwich method; therefore, it is characterized by the specific measurement of mature antibodies [9]. In contrast, Sysmex’s anti-RBD IgG antibody is measured based on a chemiluminescent enzyme immunoassay established by the conventional method [22]. This difference may have been responsible for the interesting correlation between each time point after three doses and the correlation with each NAb. Compared to anti-RBD IgG, the levels of mature anti-RBD total Ig antibodies increased slowly at two weeks after the first dose (total Ig; median 25.4 (IQR 8.5–52.1) BAU/mL, IgG; median 140.4 BAU/mL (IQR 55.4–224.3), but began to decrease slowly two months after the second dose. The levels then increased rapidly after the booster dose. Therefore, anti-RBD total Ig differently correlates with anti-RBD IgG at each time point, and the correlation with NAb is weak compared to that of anti-RBD IgG, however, the increase is particularly large in the booster dose. This phenomenon is likely due to Roche’s anti-RBD total Ig reagent, which measures only mature antibodies with high avidity. An avidity index is not included in the measurement of anti-RBD IgG and NAb.

NAb varies according to the mutant strain and recombinant, and the measurement method of NAb is complicated and not easily comparable between laboratories, whereas anti-RBD antibodies measured by automated analyzers are easy to use. However, even with the commercial assays used in this study, there are differences in methods of antibody titer measurement, or whether the factor of avidity is also considered; therefore, it is difficult to harmonize programs across different clinical laboratories. In addition, when the measurement targets are different, it is not preferable to harmonize them into one category. Rather, if the characteristics of each reagent are understood and used, it may be possible to measure humoral immunity, including avidity, by comparing the measurement reagents. This report is valuable as it explores the detailed response of humoral immunity at each time point after three doses.

Nakagama et al. [23] followed the convalescent phase of patients with COVID-19, measuring anti-RBD antibody titers using Elecsys^®^ Anti-SARS-CoV-2S (anti-RBD total Ig; Roche) and ARCHITECT SARS-CoV-2 IgG II Quantitative (Abbott) and indicated that the antibody titers measured by the Roche system were high avidity indices, and persisted even in the late convalescent period. Matusali et al. [24] investigated the dynamic association among binding and functional antibodies in healthcare workers receiving the BNT162b2 mRNA COVID-19 vaccine. They compared anti-RBD using ARCHITECT SARS-CoV-2 IgG II Quantitative, and anti-Trimeric S-IgG by LIAISON SARS-CoV-2 TrimericS IgG (DiaSorin S.p.A.) and NAb. They reported a steep decay of both anti-RBD antibodies after peak antibody titers.

Moriyama et al. [25] discovered that the quality of NAb (neutralizing specific activity/cross-reactivity) in plasma from convalescent patients with COVID-19 improved over time and may respond to variant strains. This phenomenon is known as the affinity maturation phenomenon [26]. When the time course of the antibodies against the variant strain was quantified, the neutralization activity (specific neutralization activity) and cross-reactivity (neutralization specific activity against the variant strain) per antibody increased with time. It is thought that humoral immunity is adaptable and antibodies with a high binding affinity for a circulating antigen are selected by maturation. Roche’s anti-RBD total Ig possibly measures antibodies with high binding affinity.

The antibody titer wanes over time after the second dose. The negative correlation with age, shown in Figure 2, Figure 3 and Figure 4, was reproduced in Japan and supports the findings of previous studies of different populations [3,27,28,29]. Gilbert et al. [30] compared antibody titers with the number of infected people to determine whether higher titers were associated with lower infection rates. As a high antibody titer has a preventive effect, measuring NAb, or anti-RBD antibodies with a good correlation to neutralizing antibodies is an effective method to predict the protection afforded to each individual [31,32,33]. It has also been reported that the NAb of the Omicron strain increased 97-fold one month after all three vaccine doses, compared to approximately six months after the second dose [34].

In addition, we investigated factors affecting NAb after the booster dose, such as immune parameters, laboratory tests, adverse reactions, and other viral antibody titers. Previous studies have not clearly explained the factors associated with the significant recovery of the immune response by the booster dose [35,36,37]. In this study, no significant association was observed with gender, age, adverse reactions, or other viral antibody titers for elevated NAb by the booster dose, but a significant association was observed with the behavior of immune parameters after the second dose. It was suggested that maximum humoral immunity and the maintenance of cell-mediated immunity after the second dose contributed to the effect of the booster dose. We also detected the association of low eosinophil (%) and high erythrocyte count in the phase of waning from the peak of NAb after the second dose. It has been reported that lower eosinophil counts are related to COVID-19, especially severe COVID-19 [38], but we are not aware of any reports of their association with vaccine dose. Verification by replication studies is required in the future to include a high erythrocyte count.

Regarding cell-mediated immunity, T-SPOT numbers remained higher than the cutoff value even after a lapse of time after a vaccine dose and even if there were individual differences. They also increased significantly with the booster dose. Kruse et al. [17] observed T-SPOT reactivity longer than anti-N IgG, after COVID-19 infection. Canete et al. [39] also stated that antibody titers decline after infection, but cell-mediated immunity continues for a long time. No correlation was observed between the T-SPOT numbers and the antibody titers. Interestingly, no correlation in the T-SPOT was observed with gender, but a weak positive correlation with age. This is contrary to the result that antibody titers decrease with age, and humoral immunity and cell-mediated immunity can be regarded as complementary to each other. Therefore, it was considered that immunity to COVID-19 cannot be evaluated only by the easily measured antibody titer. Most procedures for assessing cell-mediated immunity are not easily performed in most clinical laboratories. Furthermore, in the analysis of cell-mediated immunity using T-SPOT, the choice of peptide pool may affect the T-cell response. Therefore, there is a need for the development and harmonization of cell-mediated immunity testing procedures that can be performed in clinical laboratories.

A total population cohort study conducted in Sweden investigated the infection prevention effect and the aggravation prevention effect of the vaccine. According to the report, BNT162b2 efficacy decreased to 47% four to six months after the second dose, and no significant efficacy was observed after seven months [40]. This may vary by country, race, and epidemic strain of the virus—the relationship between the host and the virus. This is because the infection rate differs depending on the virus spread rate and infection prevention measures, such as using masks. However, the evidence that vaccine efficacy is lower in males than in females, and in elder people than in younger people, does not appear to be a major discrepancy between cohort study results and immunocompetence measurements. This indicates that the measurement of humoral and cell-mediated immunity in clinical laboratories is a potentially useful method of understanding the social immunity status.

The limitations of our study were the small number of subjects examined, only the BNT162b2 vaccine was studied, and the delay in starting the vaccine dose led to delayed presentation of the results. In addition, the prevalent mutant strains are changing steadily. There are many cases where it cannot be judged only by the measurement method at the time of planning in May 2021. However, as the immunity due to the mRNA vaccines does not change significantly, the study results may be considered a representation of the general transition of immunity. Our follow-up studies on humoral response and cell-mediated immunity are still ongoing. Finally, there were only a few subjects with a BMI greater than 30 or illnesses connected to obesity, old age, and medical history, all of which are thought to affect immune behavior. Therefore, it was not possible to obtain a sample size that could withstand the analysis. In addition, because the participants in the study were medical professionals, there were almost no subjects over the age of 60 due to retirement. It is hoped that a large-scale cohort will be investigated in the future based on the basic data of this study.

## 5. Conclusions

This study highlights the following findings: (1) anti-RBD total Ig was significantly increased by three doses of the BNT162b2 vaccine compared to anti-RBD IgG and NAb; (2) it was suggested that the difference between humoral immunity and cell-mediated immunity with age are complementary to each other, and it was reconfirmed that immunity to COVID-19 cannot be evaluated by antibody titer alone; (3) anti-RBD total Ig with high avidity and anti-RBD IgG were correlated well at each time point after vaccine doses; and (4) since each measurement reagent has its own characteristics, it is necessary to harmonize reagents categorized by the same characteristics, rather than harmonizing them all together as an anti-RBD antibody.

## Figures and Tables

**Table 1:** Characteristics of the health workers participating in this study.

Characteristics		Data
Age-years, Median (IQR, range)	Female (n = 31)	48 (32–56, 23–59)
	Male (n = 19)	42 (35–45, 24–63)
Job—no. (%)	Nurse	12 (24)
	Physician	2 (4)
	Medical laboratory scientist	20 (40)
	Medical office staff	16 (32)
Vaccine adverse reactions at 2 doses—no. (%)	Fever (238 °C)	7 (14)
	Pain at the injection site	46 (92)
	Fatigue/tiredness	35 (70)
	Headache	12 (24)
Vaccine adverse reactions at 3 doses—no. (%)	Fever (238 °C)	14 (28)
	Pain at the injection site	43 (86)
	Fatigue/tiredness	38 (76)
	Headache	24 (48)
Antibody (IgG) titers for another virus-COI * median (IQR)	Chickenpox	17.6 (9.9–23.6)
	Rubella	17.8 (8.8–33.7)
	Measles	16.5 (111–31.3)
	Mumps	5.3 (3.3–8.2)

* COI (cutoff index) ≥ 2.0 is set as IgG positive.

**Table 2:** Associations between levels of neutralizing antibodies at two weeks after the booster dose and results of clinical laboratory tests and immune parameters at all time points after two doses.

Factor (Unit)	Time Point after 2 Doses	NAb < 65% Inhibition Group: n = 25 Median (IQR)	NAb ≥ 65% Inhibition Group: n = 24 Median (IQR)	*p*-Value	Odds Ratio (95% Cl)
Total Ig (BAU/mL)	2 weeks (peak)	7.6 (7.0–7.9) *	8.3 (7.9–8.4) *	0.0085	18.8 (2.12–167)
T-SPOT (2.5 × 10^5^/well)	6 months	11 (6–20)	14 (8–41)	0.011	1.13 (1.03–1.24)
Eosinophils (%)	2 months	3.1 (1.7–6.1)	1.9 (1.2–3.1)	0.048	0.4 (0.17–0.99)
Red blood cells (×10^6^/μL)	2 months	4.41 (4.26–4.70)	4.73 (4.52–5.11)	0.048	1.03 (1.00–1.07)

Factors affecting levels of neutralizing antibodies (NAb) after the booster dose were analyzed using multiple logistic regression analysis. For the anti-RBD antibodies (total Ig and IgG), we adopted the explanatory variables that were most closely associated using mono-logistic analysis at all time points after one and two doses to avoid multicollinearity. * As the behavior range of the SARS-CoV-2 specific antibody is wide, the log conversion value was used as an explanatory variable. IQR, interquartile range.

## Data Availability

The data used and/or analyzed during the current study are available, only for sections non-infringing personal information, from the corresponding author on a reasonable request.

## Supplementary Materials

The following supporting information can be downloaded at: https://www.mdpi.com/article/10.3390/vaccines10071050/s1, Table S1. Outline of the research schedule of the acquired experimental data. Table S2. List of acquired clinical laboratory items for this study. Table S3. Association between neutralizing antibodies at two weeks after the booster dose and the delta from the peak of immune parameters after the second dose. Figure S1. A one-year time course of the humoral and cell-mediated immunity against SARS-CoV-2 with three doses of the BNT162b2 vaccine in all of the study subjects, classified by gender and age; Figure S2. Correlation at time points between T-SPOT and anti-RBD total Ig after the BNT162b2 vaccine; Figure S3. Correlation at time points between T-SPOT and anti-RBD IgG after BNT162b2 vaccine; Figure S4. Correlation at time points between T-SPOT and neutralizing antibodies after BNT162b2 vaccine.
