# Supplementary material for: Differential Dynamics of Humoral and Cell-Mediated Immunity with Three Doses of BNT162b2 SARS-CoV-2 Vaccine in Healthcare Workers in Japan: A Prospective Cohort Study
Source: Vaccines (Basel). 2022 Jun 30;10(7):1050. doi: 10.3390/vaccines10071050 (PMC9323262; doi:10.3390/vaccines10071050)
Supplement: Supplementary file 1 [file vaccines-10-01050-s001.zip › vaccines-1770433-supplementary.pdf]

Supplemental Tables

Table S1. Outline of the research schedule of the acquired experimental data

Time point	pre	1st vaccine dose	3 days	2 weeks	2nd vaccine dose	3 days	2 weeks	1 month	2 months	4 months	6 months	10 months	3rd (booster) vaccine dose	2 weeks
Day	-5	0	3	14	21	24	35	51	81	141	201	321	323	337
Subjects' information	●													
Medical check sheets	●		●	●		●	●	●	●	●	●	●		●
Laboratory testing	●		●	●		●	●		●		●	●		●
SARS-CoV-2 specific antibodies	●		●	●		●	●	●	●	●	●	●		●
Neutralizing antibodies against SARS-CoV-2	●		●	●		●	●	●	●	●	●	●		●
Cell-mediated immunity against SARS-CoV-2	●			●			●		●		●	●		●

Black circles indicate the timing of acquisition of specimen collection and information.

Table S2. List of acquired clinical laboratory items for this study.

Category for clinical laboratory analysis	Item name
Biochemical tests	urea nitrogen
	creatinine
	uric acid
	total bilirubin
	triglyceride
	total cholesterol
	lactate dehydrogenase
	aspartate aminotransferase
	alanine aminotransferase
	creatine kinase
	alkaline phosphatase
	γ -glutamyl transferase
	cholinesterase
	amylase
	total protein
	albumin
	glycated albumin
	c-reactive protein
	rheumatoid factor
	immunoglobulin G
	immunoglobulin A
	immunoglobulin M
	β 2 -microglobulin
	soluble interleukin-2 receptor
Immunochemical tests	Krebs von den Lungen-6
	thymus and activation-regulated chemokine
	interleukin 6
Hematology tests	leukocyte
	erythrocyte

Hematology tests	hemoglobin
	hematocrit
	platelet
	mean corpuscular volume
	mean corpuscular hemoglobin
	mean corpuscular hemoglobin concentration
	neutrophil
	eosinophil
	basophil
	lymphocyte
	monocyte

Table S3. Association between neutralizing antibodies at two weeks after the booster dose and the delta from the peak of immune parameters after the second dose.

Factor	Time points after 2nd vaccination	< 65% NAb inhibition group: n = 25 Median (IQR)	≥ 65% NAb inhibition group: n = 24 Median (IQR)	*p*-value	Odds Ratio (95% CI)
Total Ig (BAU/mL)	1 month	Δ-0.4 (-0.5 ~ -0.2)	Δ-0.5 (-0.6 ~ -0.4)	0.069	0.029 (0.00063–1.32)
IgG (BAU/mL)		Δ-0.3 (-0.4 ~ -0.3)	Δ-0.3 (-0.4 ~ -0.2)	0.55	2.47 (0.13–45.8)
Neutralizing Ab (%)		Δ-13.7 (-16.8 ~ -7.0)	Δ-10.7 (-15.8 ~ -6.6)	0.56	1.02 (0.95–1.11)
Total Ig (BAU/mL)	2 months	Δ-0.6 (-0.8 ~ -0.4)	Δ-0.8 (-1.0 ~ -0.6)	0.055	0.15 (0.022–1.04)
IgG (BAU/mL)		Δ-1.1 (-1.3 ~ -0.9)	Δ-1.1 (-1.4 ~ -0.9)	0.50	0.51 (0.072–3.61)
Neutralizing Ab (%)		Δ-50.1 (-55.9 ~ -34.1)	Δ-47.3 (-56.6 ~ -42.2)	0.63	0.99 (0.95–1.03)
Total Ig (BAU/mL)	4 months	Δ-1.0 (-1.3 ~ -0.7)	Δ-1.2 (-1.6 ~ -1.0)	**0.044**	**0.27 (0.077–0.96)**
IgG (BAU/mL)		Δ-1.9 (-2.2 ~ -1.6)	Δ-2.0 (-2.3 ~ -1.9)	0.19	0.40 (0.10–1.57)
Neutralizing Ab (%)		Δ-51.3 (-63.3 ~ -31.7)	Δ-55.9 (-65.2 ~ -49.7)	0.081	0.96 (0.93–1.00)
Total Ig (BAU/mL)	6 months	Δ-1.2 (-1.4 ~ -0.8)	Δ-1.4 (-1.7 ~ -1.1)	0.084	0.37 (0.12–1.14)
IgG (BAU/mL)		Δ-2.4 (-2.8 ~ -2.2)	Δ-2.6 (-2.9 ~ -2.3)	0.28	0.53 (0.17–1.66)
Neutralizing Ab (%)		Δ-65.4 (-69.9 ~ -39.5)	Δ-71.1 (-76.8 ~ -67.5)	**0.031**	**0.95 (0.91–1.00)**
Total Ig (BAU/mL)	10 months	Δ-1.5 (-1.8 ~ -1.1)	Δ-1.6 (-1.9 ~ -1.4)	0.31	0.61 (0.24–1.59)
IgG (BAU/mL)		Δ-2.8 (-3.3 ~ -2.7)	Δ-3.0 (-3.3 ~ -2.7)	0.64	0.79 (0.29–2.15)
Neutralizing Ab (%)		Δ-64.7 (-68.1 ~ -41.4)	Δ-72.5 (-78.4 ~ -63.6)	**0.032**	**0.95 (0.91–1.00)**

As the behavior range of anti-receptor-binding domain antibodies (total Ig and IgG) is wide, the log conversion value was used as an explanatory variable. Δ, Difference between the peak after the second dose and each time point.
Bold indicate $p < 0.05$; NAb, neutralizing antibodies

Supplemental Figures

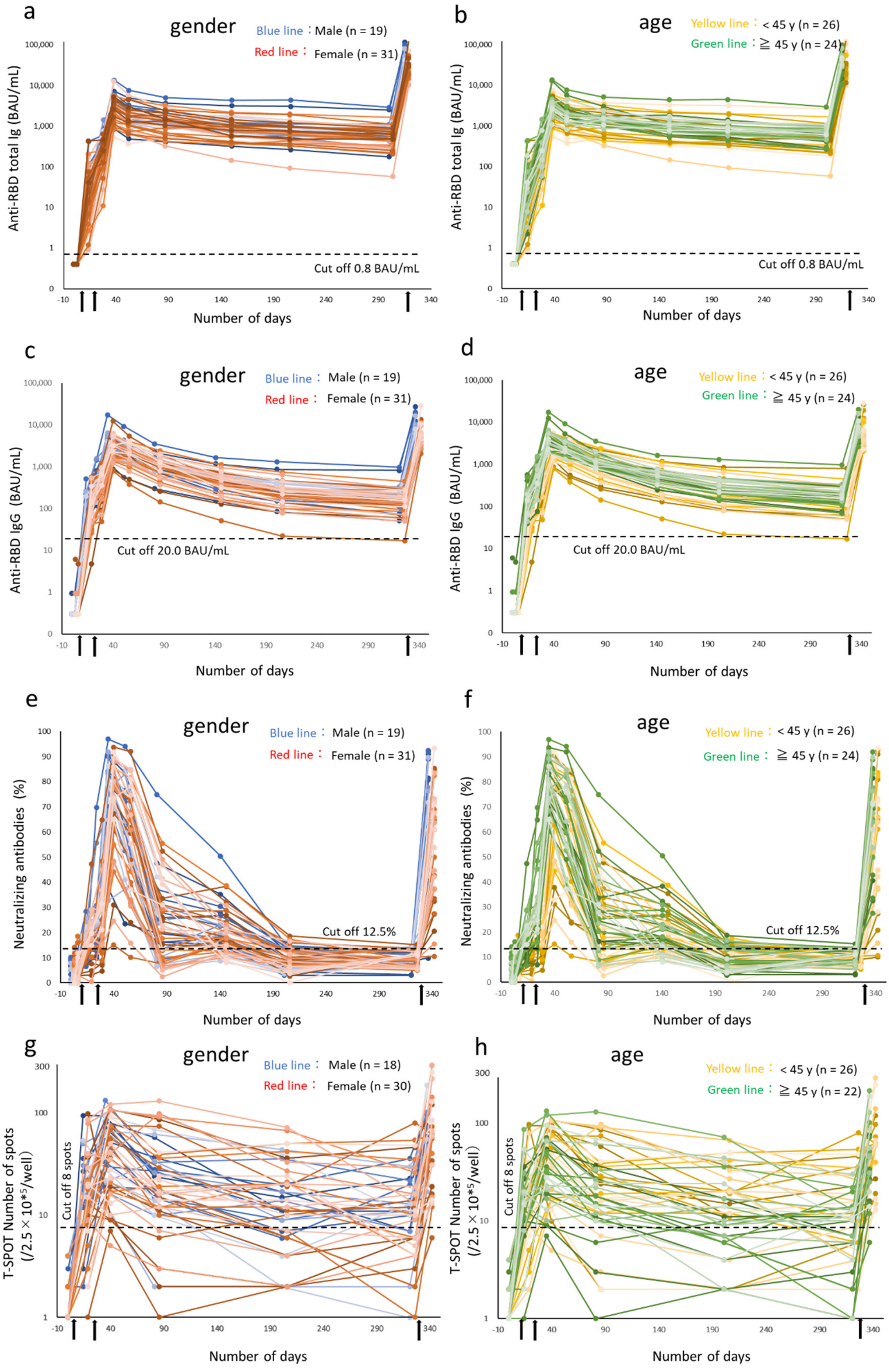

Figure S1. (a-h). A one-year time course of the humoral and cell-mediated immunity against SARS-COV-2 with three doses of the BNT162b2 vaccine in all of the study subjects, classified by gender and age.

Individual's levels of the anti-RBD total Ig (a, b) and IgG (c, d), neutralizing antibodies (e, f), and T-SPOT (g, h) are represented by a polygonal line in time series after vaccination. Blue and red lines represent male and female, respectively. Green and yellow lines represent subjects above and below 45 years old, respectively. Dashed line represents the cutoff value in each immunity parameter. X-axis: number of days at the time series based on the first dose (0 days), and the arrow (black) indicates the vaccination date. Y-axis: a and b, anti-RBD total Ig (Elecsys® anti-SARS-CoV-2 S; Roche); c and d, anti-RBD IgG (HISCL™ SARS-CoV-2 S-IgG; Sysmex); e and f, neutralizing antibody (SARS-CoV-2 Neutralization Antibody Detection Kit; MBL), g, h; T-SPOT (T-SPOT® Discovery SARS-CoV-2; Oxford Immunotec). Anti-RBD antibodies and neutralizing antibodies were shown on a log scale.

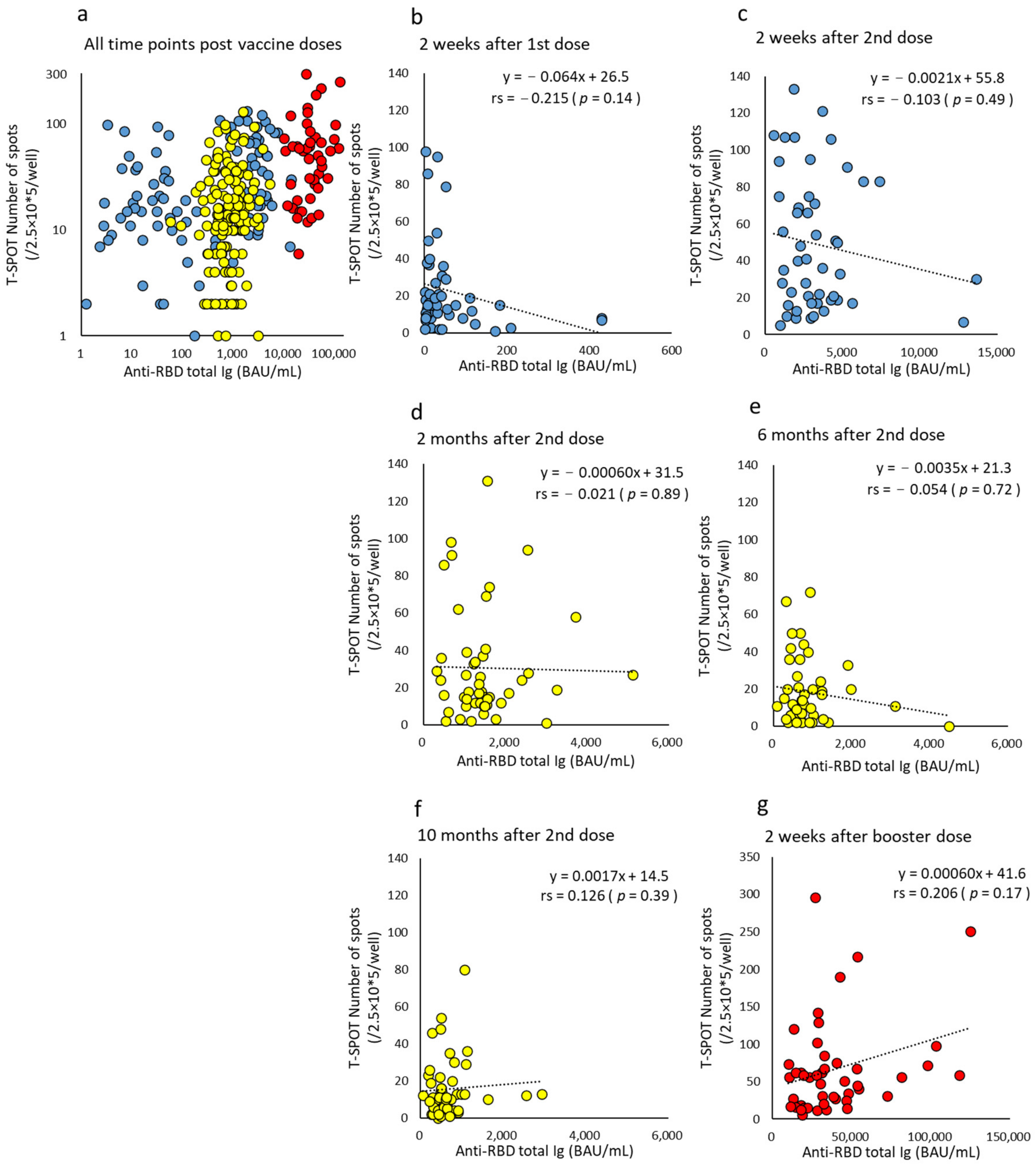

Figure S2. (a-g). Correlation at time points between T-SPOT and anti-RBD total Ig after BNT162b2 vaccine.

The levels of parameters indicating T-SPOT and anti-RBD Ig are plotted, and the correlation is compared using all or each time point post-vaccination. Antibody titers at all time points are indicated in a log scale (a). Time point at two weeks after the second dose is represented by blue (b, c), from two months to ten months after the second dose is in yellow (d-f), and two weeks after the booster dose is in red (g). p -values were calculated using Spearman's rank correlation test, and significance was set at 0.01 (rs, Spearman's rank correlation coefficient).

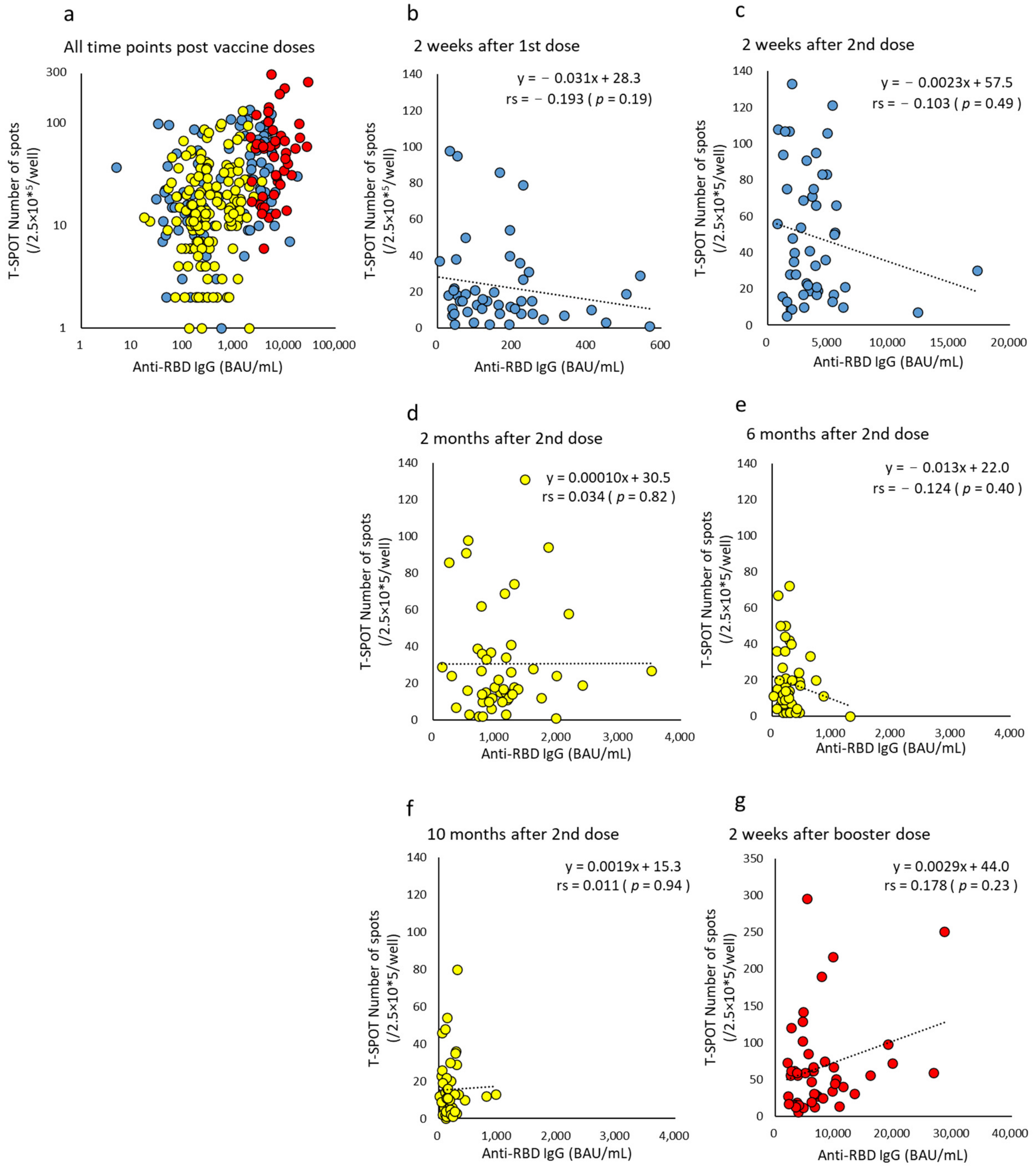

Figure S3. (a-g). Correlation at time points between T-SPOT and anti-RBD IgG after BNT162b2 vaccine.

The levels of parameters indicating T-SPOT and anti-RBD IgG are plotted, and the correlation is compared using all or each time point post-vaccination. Antibody titers at all time points are indicated in a log scale (a). Time point at two weeks after the second dose is represented by blue (b, c), from two months to ten months after the second dose is in yellow (d-f), and two weeks after the booster dose is in red (g). *p* -values were calculated using Spearman's rank correlation test, and significance was set at 0.01 (rs, Spearman's rank correlation coefficient).

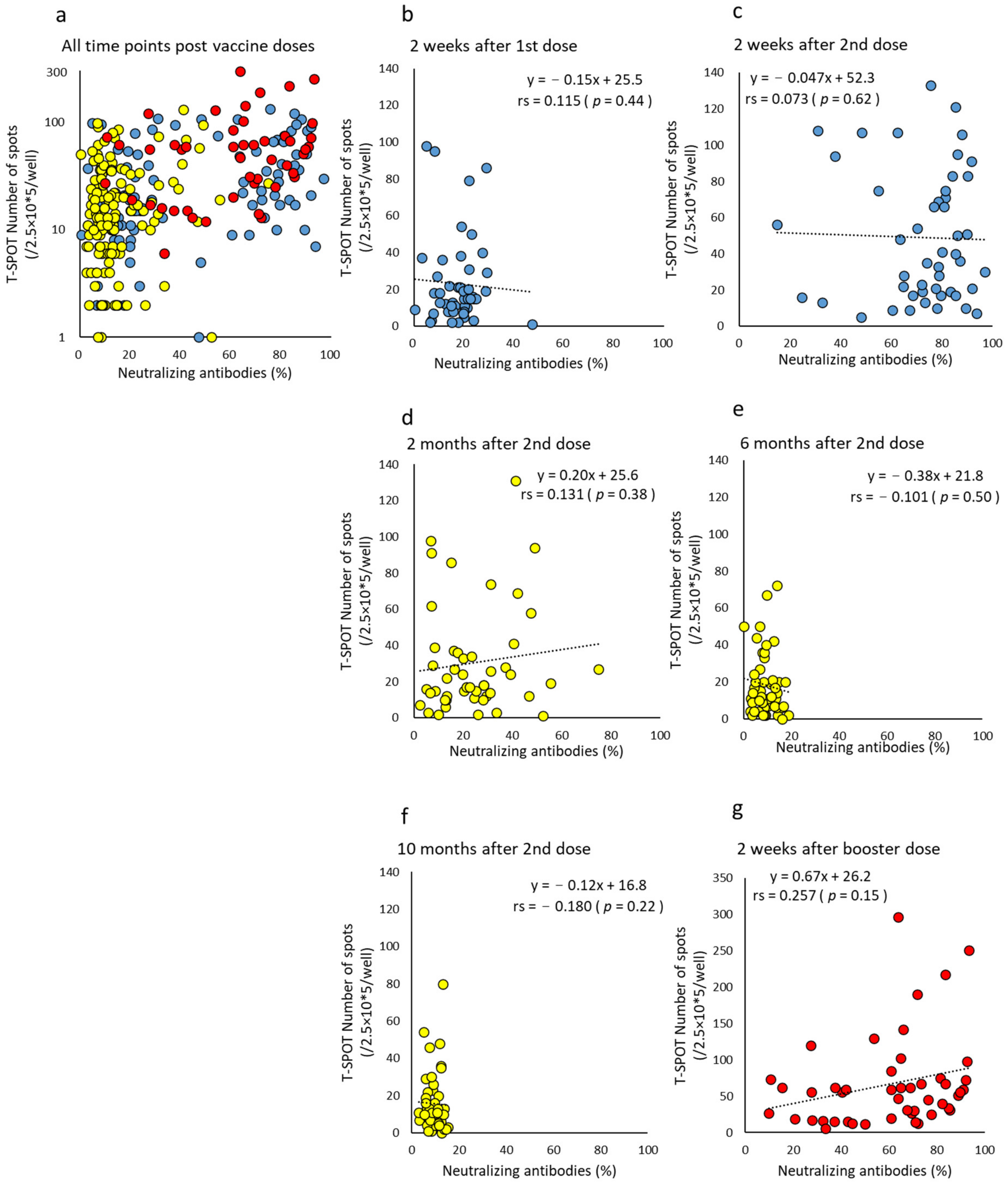

Figure S4. (a-g). Correlation at time points between T-SPOT and neutralizing antibodies after BNT162b2 vaccine.

The levels of parameters indicating T-SPOT and neutralizing antibodies are plotted, and the correlation is compared using all or each time point post-vaccination. Antibody titers at all time points are indicated in a log scale (a). Time point at two weeks after the second dose is represented by blue (b, c), from two months to ten months after the second dose is in yellow (d-f), and two weeks after the booster dose is in red (g). *p* -values were calculated using Spearman's rank correlation test, and significance was set at 0.01 (rs, Spearman's rank correlation coefficient).
